# The Impact of the Number of Neoadjuvant Chemotherapy Cycles on Outcomes in Advanced Ovarian Cancer: A Narrative Review

**DOI:** 10.3390/cancers18040545

**Published:** 2026-02-07

**Authors:** Ana Carla Franco Ubinha, Camila Musa Honorato, Marcelo Henrique dos Santos, Luis Pires de Melo Filho, Luciano Ipólito Branquinho, Adhemar Longatto-Filho, Ricardo Dos Reis

**Affiliations:** 1 Department of Gynecologic Oncology, Barretos Cancer Hospital, Barretos 14784-400, Brazil; oncomhs@gmail.com (M.H.d.S.); luisfilho4@hotmail.com (L.P.d.M.F.); lucianoibranquinho@gmail.com (L.I.B.); drricardoreis@gmail.com (R.D.R.); 2 Barretos School of Health Sciences, Dr. Paulo Prata-FACISB, Barretos 14785-002, Brazil; camilamusa94@gmail.com; 3 Molecular Oncology Research Center, Barretos Cancer Hospital, Barretos 14784-400, Brazil; 4 Medical Laboratory of Medical Investigation, Department of Pathology, Medical School, University of São Paulo, São Paulo 01246-903, Brazil; 5 Life and Health Sciences Research Institute (ICVS), School of Medicine, University of Minho, 4710-057 Braga, Portugal; 6 ICVS/3B’s—PT Government Associate Laboratory, 4710-057 Braga, Portugal; 7 ICVS/3B’s—PT Government Associate Laboratory, 4805-017 Guimarães, Portugal

**Keywords:** ovarian cancer, cytoreduction surgery, neoadjuvant chemotherapy, interval debulking surgery

## Abstract

Although standard chemotherapy for three to four cycles followed by surgery is considered safe and effective in the management of advanced ovarian cancer, the impact of extending treatment beyond this period remains uncertain. Some authors suggest that the number of neoadjuvant chemotherapy cycles does not directly affect prognosis and may reflect a confounding bias. Others, however, indicate that a prolonged exposure to chemotherapy can promote the selection of resistant tumor clones, negatively influencing patient survival and disease progression. This review aims to summarize the current evidence on the topic, evaluating the effect of prolonged chemotherapy on surgical cytoreduction and survival. To achieve this, we conducted an analysis of the English-language literature available in PubMed, focusing on treatment duration, achievement of complete surgical resection, survival outcomes, and prognostic factors.

## Simple Summary

Ovarian cancer is the gynecologic malignancy with the highest mortality rate. Standard treatment is based on complete surgical resection combined with platinum-based chemotherapy. In cases of advanced disease or in patients with impaired clinical status, neoadjuvant chemotherapy may be considered as a therapeutic strategy, followed by interval debulking surgery. Classic studies recommend the administration of three to four cycles of chemotherapy before surgical intervention. However, in real-world practice, this number is often exceeded, and the literature remains inconclusive regarding the relationship between the number of neoadjuvant cycles and oncologic outcomes.

## 1. Introduction

Ovarian cancer is the eighth most common malignancy among women worldwide and remains the gynecologic cancer with the highest mortality rate [1]. Epithelial tumors are the most prevalent, with high-grade serous carcinoma being the most common histologic subtype and characterized by aggressive biological behavior [2]. Diagnosis is often made at advanced stages, as early symptoms are nonspecific and effective screening strategies are extremely limited [3,4,5,6,7].

In advanced epithelial ovarian cancer, standard treatment consists of surgery combined with platinum-based chemotherapy. Complete surgical resection with no macroscopic residual disease is the primary objective, as residual disease is associated with poorer oncologic outcomes [8]. At a minimum, bilateral salpingo-oophorectomy, hysterectomy, and omentectomy are recommended. Additional procedures, including peritonectomy, lymphadenectomy, resection of pelvic organs, and upper abdominal or thoracic surgery, may be required according to disease extent [9].

In 1975, Griffiths was the first to demonstrate the association between the size of residual disease and oncologic survival [10]. Since then, classic evidence has shown a linear relationship between the extent of cytoreduction and survival, establishing the absence of residual disease as the main prognostic factor in ovarian cancer [11,12]. Bristow et al. further demonstrated that each 10% increase in maximal cytoreduction results in an approximate 5.5% improvement in median survival [11].

In cases of high disease volume, tumor unresectability, or unfavorable clinical conditions, neoadjuvant chemotherapy (NACT) followed by interval debulking surgery (IDS) and subsequent adjuvant chemotherapy is a therapeutic strategy that has been increasingly adopted and supported by the literature [8,13,14].

Five major randomized clinical trials compared upfront primary debulking surgery (PDS) plus adjuvant chemotherapy with NACT followed by IDS in advanced ovarian cancer [9,15,16,17,18,19]. In all studies, NACT was limited to three or four cycles. In real-world practice, however, more than four cycles are frequently administered, particularly in patients with a high tumor burden, partial response to initial therapy, or clinical and social barriers to early surgery [20].

Observational and retrospective studies show a high variability, and the literature remains inconclusive regarding the number of NACT cycles and oncologic outcomes. This review examines the rationale for NACT in ovarian cancer and the evidence on prolonged neoadjuvant therapy and patient survival.

## 2. Principles of Neoadjuvant Therapy in Ovarian Cancer

The first major randomized trial evaluating neoadjuvant therapy for advanced ovarian cancer was published in 2010 by Vergote et al. (EORTC 55971). A total of 632 patients with FIGO stage IIIC or IV disease were randomized to PDS with adjuvant chemotherapy or NACT (three cycles) followed by IDS and subsequent adjuvant chemotherapy. Median overall survival (OS) was similar between groups (29 vs. 30 months), as was median progression-free survival (PFS) (12 months in both), confirming the non-inferiority of NACT. The complete resection of all disease remained the most important independent prognostic factor. Complete cytoreduction was achieved in 19.4% of the PDS group and 51.2% of the NACT group [15].

In 2015, the CHORUS study analyzed 550 patients with FIGO stage III or IV epithelial ovarian cancer randomized to PDS with adjuvant chemotherapy or NACT (three platinum-based cycles) followed by IDS and subsequent adjuvant chemotherapy. Median OS was 22.6 months in the PDS group and 24.1 months in the NACT group, while median PFS was similar between groups (10.7 vs. 12.0 months, respectively), again demonstrating the non-inferiority of the neoadjuvant strategy. The NACT arm also showed lower surgical morbidity and perioperative mortality. Complete cytoreduction was achieved in 17% of the PDS group and 39% of the NACT group. Residual disease volume remained a key prognostic factor in both groups [16].

An integrated analysis by Vergote et al. combined individual patient data from the EORTC 55971 and CHORUS trials. With a similar OS and PFS between the two groups, the neoadjuvant approach is considered a safe and effective alternative for advanced ovarian cancer, particularly in patients with extensive disease or high surgical risk, without compromising clinical outcomes [21].

The Japanese JCOG 0602 study also compared PDS with adjuvant chemotherapy versus NACT (four cycles) followed by IDS in 301 patients with advanced ovarian cancer (FIGO stage III–IV). The 2016 publication focused on surgical invasiveness and adverse events, showing that neoadjuvant therapy could reduce operative time, blood loss, extensive procedures, and postoperative complications [17]. The 2020 report provided oncologic outcomes, with a median OS of 49 months for PDS and 44.3 months for IDS, with no statistically significant difference. Median PFS was 15.1 months for PDS and 16.4 months for the NACT group, confirming the non-inferiority of the neoadjuvant strategy. Complete cytoreduction was achieved in 12% of the PDS group and 64% of the NACT group [18].

Table 1 summarizes the key findings from the EORTC 55971, CHORUS, and JCOG 0602 trials.

However, shortly after the publication of these studies, several questions were raised in the literature. First, the included population was highly heterogeneous, including patients with different clinical stages (IIIB, IIIC, and IV), different histologies, and a wide range of performance statuses. Second, complete cytoreduction rates were relatively low in these studies, especially in the upfront surgery group. Finally, absolute OS and PFS values remained low, despite being similar between groups, and were lower than those reported in contemporary series from high-volume cancer centers [9,12].

The SCORPION study, a randomized clinical trial published in 2020, was designed to overcome some of the limitations of previous trials, including population heterogeneity and low surgical standardization. Eighty-four patients were analyzed in the PDS group and 74 in the NACT group (three or four cycles), with the overall population consisting of patients with advanced ovarian cancer, FIGO stage IIIC–IV. Complete cytoreduction rates were 47.6% in the PDS group and 67% in the IDS group, while median OS was 41 months in the PDS group and 43 months in the IDS group. Median PFS was 15 months in the PDS group and 14 months in the IDS group, respectively. In the subgroup analysis of patients with complete cytoreduction, there was no statistically significant difference in oncologic outcomes between primary and interval surgery. Moreover, interval surgery was associated with lower postoperative complications, including mortality. Thus, this study concluded that NACT is as effective as PDS, but with different toxicity profiles [19].

The TRUST trial was designed to overcome the limitations of earlier studies and to answer the key question of the optimal timing for cytoreductive surgery in advanced ovarian cancer. The study focused on achieving a high rate of complete resections with no visible residual disease, allowing a direct comparison between primary and interval complete cytoreduction. A major strength of the trial was that all surgeries were performed in high-volume cancer centers by surgeons who had proven their expertise [9].

The results of the TRUST trial were presented at the 2025 annual meeting of the American Society of Clinical Oncology (ASCO) but have not yet been published in a peer-reviewed journal. This phase III multicenter trial included 688 eligible patients with advanced ovarian cancer (FIGO stage IIIB–IVB) and good performance status (ECOG 0–1), comparing PDS with NACT followed by IDS. The primary endpoint was OS. Median OS was 54.3 months in the PDS arm versus 48.3 months in the NACT arm, a numerical difference that did not reach statistical significance. Median PFS was significantly higher with PDS (22.2 vs. 19.7 months). Also, the PFS was significantly higher in Stage III and in patients with complete gross resection. This is the first trial to demonstrate a PFS benefit in patients undergoing PDS compared with IDS. Postoperative mortality was <1%, demonstrating the safety of surgery in specialized centers [22].

Table 2 summarizes the key findings from the SCORPION and TRUST trials.

In the same line of investigation, the SUROVA study was recently published as a large international retrospective analysis evaluating PDS versus NACT followed by IDS in patients with advanced epithelial ovarian cancer (FIGO stage IIIB–IVB). The study included 3286 patients, and, to account for differences in baseline patient profiles, a propensity score matching approach was applied using seven clinical and pathological variables. Among patients receiving NATC, the median number of cycles was three. After matching, OS was comparable between treatment strategies, with no statistically significant difference observed. The most favorable results were observed in patients undergoing PDS with complete cytoreduction and no complications [23].

The evidence presented leads to the following conclusions: (1) complete tumor resection is strongly associated with superior oncologic outcomes; and (2) when upfront PDS is not feasible, NACT followed by IDS represents a valid alternative without compromising survival outcomes. These conclusions are corroborated by a systematic review by Coleridge et al., published in 2021 in the Cochrane Library, which found no significant differences in survival between primary surgery and NACT, while reporting lower perioperative morbidity with NACT [24].

However, a remaining question is whether the results of neoadjuvant trials can be extrapolated to scenarios in which more than four NACT cycles are commonly administered.

## 3. Impact of the Number of NACT Cycles on Oncologic Outcomes

Although the available clinical trials demonstrate that NACT followed by IDS does not compromise survival outcomes compared with PDS, the extrapolation of these results to scenarios in which a higher number of NACT cycles is administered remains uncertain.

**Figure 1:**
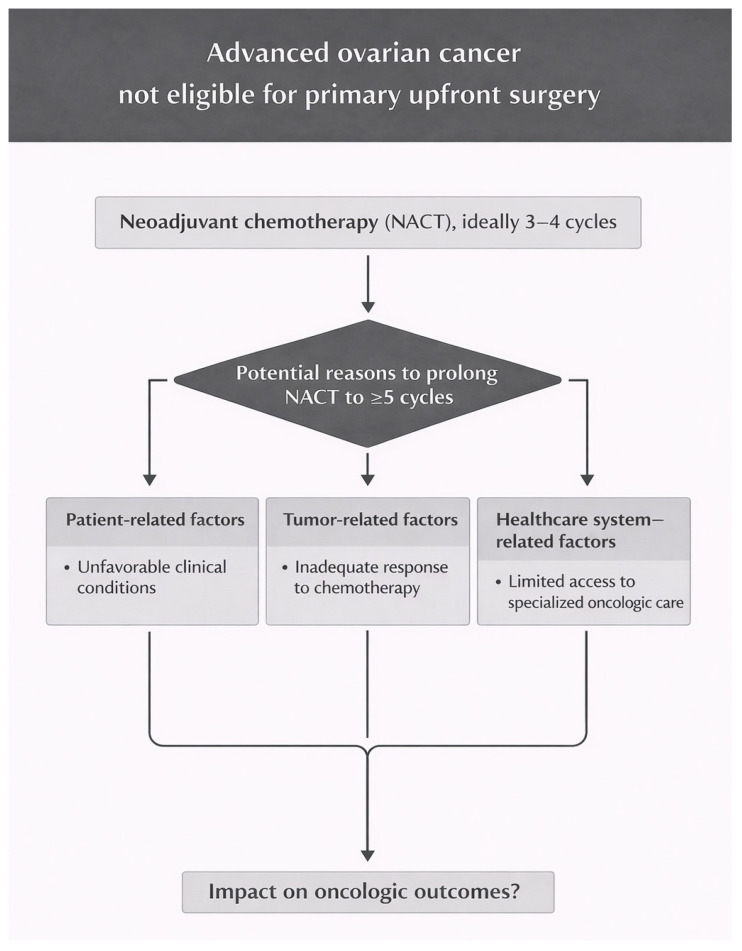
Potential reasons for prolonged neoadjuvant chemotherapy in real-world ovarian cancer management.

Defining the optimal number of NACT cycles persists as a challenge [25]. Multiple factors may justify prolonged neoadjuvant chemotherapy and delayed surgery, including patient characteristics, tumor features, and healthcare access, as illustrated in Figure 1 [20,25]. Individual clinical factors such as age, comorbidities, and performance status, as well as tumor factors including disease extent and response to chemotherapy, directly influence the indication and duration of neoadjuvant treatment. Furthermore, the availability of specialized oncology teams and healthcare resources plays a decisive role in clinical decision, contributing to significant variations in practice and, in many cases, to delayed IDS [20,25].

The lack of consensus in the literature regarding the optimal number of NACT cycles reflects the methodological heterogeneity of available studies, which are predominantly retrospective, with limited prospective data and no randomized clinical trials. Furthermore, analyses vary widely in tumor characteristics, the number of cycles administered prior to IDS, and the surgical parameters assessed, contributing to conflicting results in survival outcome.

This section contrasts studies showing no independent impact of the number of NACT cycles on oncologic outcomes with those suggesting a negative effect of prolonged NACT.

### 3.1. Studies Reporting No Direct Impact of the Number of NACT Cycles on Oncologic Outcomes

According to some authors, the number of NACT cycles does not have a direct impact on oncologic outcomes. These studies, summarized in Table 3 in chronological order, demonstrated that the main prognostic determinants were complete surgical cytoreduction, chemotherapy response, and residual disease, rather than the absolute number of cycles [26,27,28,29,30,31,32,33,34,35,36,37,38,39,40,41,42,43].

The need for additional cycles of neoadjuvant chemotherapy is closely linked to underlying tumor biology and behavior. More aggressive tumors and those less responsive to systemic therapy are more likely to require prolonged NACT in order to achieve sufficient disease control prior to IDS. Some evidence suggests that selected patients may benefit from additional cycles, which could reduce surgical morbidity, provided complete resection is achieved.

Plett et al. (2020) published a multicenter retrospective study on the role of prolonged NACT beyond five cycles in advanced ovarian cancer [38]. A total of 308 patients were analyzed, and the number of chemotherapy cycles did not affect OS or PFS. Conversely, patients achieving complete cytoreduction had a longer survival compared with those with residual disease [38].

Marchetti et al. (2021) conducted a retrospective study using propensity score matching to assess the impact of the number of NACT cycles on oncologic outcomes [39]. After matching patients with similar clinical and tumor characteristics, groups receiving up to four cycles were compared with those receiving five or more cycles prior to IDS. No significant differences in OS or PFS were observed between the groups, provided complete cytoreduction was achieved. These findings suggest that, after controlling for potential confounders, the number of NACT cycles does not have an independent prognostic impact [39].

Perrone et al. (2023) further emphasized the importance of complete cytoreduction in patients undergoing prolonged NACT [43]. In this retrospective study, 286 patients were included: 100 underwent IDS after up to four NACT cycles, while 186 underwent surgery after five or more cycles. Both PFS and OS did not differ significantly between groups when complete cytoreduction was achieved, indicating that delayed surgery does not compromise oncologic outcomes if maximal cytoreduction is obtained. However, the presence of residual disease after surgery, even minimal, was associated with worse prognosis and remained the determinant of survival [43].

Thus, complete cytoreduction, with no visible residual disease, remains the primary prognostic factor and is consistently associated with the best oncologic outcomes [11,12,44]. This represents the central finding of studies reporting no independent impact of the number of NACT cycles.

### 3.2. Studies Reporting Negative Direct Impact of the Number of NACT Cycles on Oncologic Outcomes

Other studies have reported a direct association between a higher number of NACT cycles and poorer oncologic outcomes. These studies are summarized in Table 4 in chronological order [20,45,46,47,48,49,50,51]. Potential reasons underlying these unfavorable outcomes are discussed below.

In the meta-analysis by Bristol and Chi, which included 835 patients, each additional preoperative chemotherapy cycle was associated with a 4.1-month reduction in median survival (*p* = 0.046). These findings suggest that an excessive extension of NACT may compromise oncologic outcomes, highlighting the importance of performing complete cytoreductive surgery without delay [45].

Mathematical models of tumor evolution suggest that a prolonged exposure to chemotherapy can promote the selection of resistant cells, providing a biological rationale for strategies that prioritize early tumor reduction with maximal cytoreduction in ovarian cancer [52]. This concept is further supported by the Goldie–Coldman hypothesis, which proposes that heterogeneous tumors contain pre-existing resistant clones whose expansion is favored by prolonged chemotherapy exposure [53]. This may represent a biological explanation for the negative impact of prolonged neoadjuvant chemotherapy.

In the study by Bogani et al. (2017), 193 patients were analyzed to evaluate the impact of the number of NACT cycles on oncologic outcomes [48]. A higher number of cycles was not associated with an increased rate of complete cytoreduction. However, patients receiving five or more NACT cycles had worse survival outcomes, which the authors attributed to potential tumor chemoresistance [48].

At Memorial Sloan Kettering Cancer Center, 199 patients undergoing IDS were analyzed. Receiving more than four NACT cycles was independently associated with poorer OS and PFS, even when complete cytoreduction was achieved. The authors highlighted that the need for extended chemotherapy reflects, from the outset, a tumor with worse prognosis, and that achieving complete cytoreduction cannot fully overcome the negative impact of prolonged NACT [20].

In the study by Thomas et al. (2022), 928 patients were included [50]. Almost half underwent delayed IDS after ≥5 NACT cycles, a practice common in the local population. Prolonged NACT did not increase the rate of complete cytoreduction (84.7% in standard IDS vs. 79.1% in delayed IDS). Patients undergoing delayed IDS had worse PFS and a trend toward lower OS, confirming that postponing surgery compromises oncologic outcomes [50].

In the multicenter study by Gaba et al. (2025), 2498 patients with ovarian cancer were analyzed, comparing three groups: (1) IDS after 3–4 NACT cycles, (2) delayed IDS after ≥5 NACT cycles, and (3) patients who did not undergo surgery after ≥5 NACT cycles [51]. Standard interval surgery showed significantly superior survival outcomes, demonstrating that complete cytoreduction achieved after prolonged NACT does not confer the same prognostic benefit as early interval debulking. However, patients who did not undergo surgery at any point had the poorest outcomes, with overall survival approximately half that of the delayed surgery group. The key strengths of the study included the large patient population, its multicenter and international design, the novel comparison between delayed surgery and no surgery, and the inclusion of diverse populations across different socioeconomic contexts. However, its retrospective design represents an important limitation [51].

Overall, meta-analyses and large retrospective cohorts generally suggest that prolonged NACT (≥4–5 cycles) may be associated with worse overall survival and, in some cases, poorer progression-free survival, even when complete or maximal cytoreduction is achieved. As discussed previously, one plausible explanation for these findings is that extended chemotherapy exposure may promote the selection or induction of chemoresistant tumor cell populations, thereby adversely affecting oncologic outcomes.

### 3.3. How Can These Data Be Interpreted?

The available evidence should be interpreted with caution, as it is largely derived from retrospective studies, with inherent limitations and bias. Nevertheless, several clinically relevant considerations can be drawn from the existing data.

First, tumors that show poor response to chemotherapy tend to exhibit more aggressive biological behavior and are consistently associated with worse prognostic outcomes. In this context, it remains unclear whether unfavorable survival results are driven by a higher number of neoadjuvant chemotherapy cycles or whether they primarily reflect underlying tumor biology. This distinction is difficult to establish in retrospective analyses and represents a major source of interpretation bias.

Second, tumors that are intrinsically resistant to chemotherapy may become further resistant with the administration of additional cycles. Prolonged exposure to systemic therapy can promote selective pressure on heterogeneous tumor cell populations, favoring the emergence of resistant clones. This provides a plausible biological explanation for the association between extended NACT and poorer oncologic outcomes reported in some studies.

Third, and equally important, advanced ovarian cancer is a heterogeneous disease with respect to clinical presentation and access to treatment. Patient-related factors, disease burden, surgical feasibility, and differences in healthcare resources may all influence prognosis. These factors further complicate the interpretation of oncologic outcomes and are also discussed below.

## 4. Other Factors Influencing Oncologic Outcomes

Beyond the number of NACT cycles and the timing of surgery, several other factors influence oncologic outcomes in advanced ovarian cancer.

Several clinical studies have investigated predictors of response to NATC in advanced ovarian cancer, demonstrating that tumor characteristics and treatment response are strongly associated with oncologic outcomes. For example, the normalization of CA-125 at the time of interval surgery correlates with higher rates of complete resection and improved PFS after NACT, suggesting that tumor sensitivity to chemotherapy reflects underlying biological behavior [54]. Extensive disease at baseline has been associated with a poorer response to NACT, further supporting the influence of baseline clinical and tumor-related factors on treatment efficacy beyond the number of chemotherapy cycles administered. Prognostic tools such as the chemotherapy response score (CRS) further demonstrate that patients achieving a higher histopathologic response after NACT experience significantly improved survival [34,55].

As previously discussed, the SUROVA study explicitly acknowledges the strong selection bias inherent to real-world treatment strategies in this setting. To address this limitation, the authors adjusted their analyses for key baseline clinical variables, including patient condition, disease burden, and surgical feasibility, and applied matching methods to compare more homogeneous patient groups. This approach aimed to reduce confounding and to better isolate the impact of treatment strategy from underlying clinical and biological differences [23].

In addition to these clinical considerations, molecular characteristics such as BRCA mutation status may influence chemosensitivity and response to systemic and targeted therapies. The use of maintenance treatments, including PARP inhibitors and bevacizumab, also plays a relevant role in overall and progression-free survival and may modify outcomes achieved after NACT and cytoreductive surgery.

The histologic subtype is another important consideration. Studies have shown that NACT does not confer a survival benefit in patients with ovarian clear cell carcinoma compared with primary surgery, although the rates of complete cytoreduction are similar between approaches [56].

Lopes et al. published a retrospective cohort study that evaluated the prognostic significance of the chemotherapy response score, post-treatment CA-125 levels, and tumor-infiltrating lymphocyte density in patients with high-grade serous ovarian carcinoma treated with six cycles of NACT followed by surgery. Among 110 eligible patients, a higher chemotherapy response score, particularly in omental specimens, was associated with improved OS. Post-treatment CA-125 levels ≤ 35 U/mL independently correlated with higher chemotherapy response score categories and favorable survival outcomes. Tumor-infiltrating lymphocyte levels were not independently associated with OS or disease-free survival [57].

## 5. International Guideline Recommendations and Future Perspectives

International guidelines recommend reassessment for IDS after approximately three cycles of NACT. This approach is indicated when there is clinical, radiologic, and biochemical response, as well as an adequate patient condition. In selected cases, additional cycles may be necessary. However, the guidelines emphasize that this decision should be individualized. There is no robust evidence supporting an oncologic benefit from routinely extending NACT [8,58].

Importantly, the optimal number of NACT cycles is also a question raised in the updated 2025 ASCO guideline. Due to the lack of randomized trials defining an ideal number of cycles, the guideline recommends that treatment decisions be based on response to therapy rather than on a fixed number of cycles. Clinical evaluation should be performed at each cycle, with serial CA-125 assessment and early radiologic reassessment, preferably after three cycles of NACT. Surgical feasibility should then be re-evaluated by a multidisciplinary team. In patients with response or stable disease and a high likelihood of achieving complete cytoreduction, IDS should be performed after no more than four cycles of NACT. The prolongation of NACT beyond this point should be considered only in selected cases, acknowledging the potential association with poorer oncologic outcomes.

Prospective clinical trials are ongoing. The GOGER (NCT02125513) and CHRONO (NCT03579394) studies are also evaluating the impact of the number of NACT cycles prior to surgery, and their results are awaited to better define the optimal therapeutic strategy in this setting [59].

## 6. Conclusions

The treatment of advanced ovarian cancer remains challenging to standardize. The disease is heterogeneous, both in clinical presentation and in access to care in real-world settings. Defining an optimal number of NACT cycles applicable to all patients is limited and also unresolved in the literature. Based on the available evidence, several practical considerations should guide clinical decision-making:

(1) Complete surgical cytoreduction should remain the primary therapeutic goal, as it is consistently associated with the best oncologic outcomes.
(2) Whenever feasible, interval debulking surgery should be planned after three to four cycles of NACT, with coordinated institutional logistics to allow timely surgery by experienced multidisciplinary teams.
(3) Prolonged NACT beyond four cycles should be reserved for carefully selected patients with a poor initial response, with the recognition that these cases often reflect unfavorable tumor biology and may be associated with increased chemoresistance and poorer oncologic outcomes.

## Figures and Tables

**Table 1:** Key findings from the EORTC 55971, CHORUS, and JCOG 0602 trials.

Trial	Analyzed Patients	FIGO Stages	Number of NACT Cycles	Complete Resection (%)	Median OS (Months)	Median PFS (Months)
EORTC 55971 [15]	632	IIIC–IV	3	PDS 19.4 NACT/IDS 51.2	PDS 29 NACT/IDS 30	PDS 12 NACT/IDS 12
CHORUS [16]	550	III–IV	3	PDS 17 NACT/IDS 39	PDS 22.6 NACT/IDS 24.1	PDS 10.7 NACT/IDS 12
JCOG 0602 [17,18]	301	III–IV	4	PDS 12 NACT/IDS 64	PDS 49 NACT/IDS 44.3	PDS 15.1 NACT/IDS 16.4

PDS, primary debulking surgery; NACT, neoadjuvant chemotherapy; IDS, interval debulking surgery; OS, overall survival; PFS, progression-free survival.

**Table 2:** Key findings from the SCORPION and TRUST trials.

Trial	Analyzed Patients	FIGO Stages	Number of NACT Cycles	Complete Resection (%)	Median OS (Months)	Median PFS (Months)
ESCORPION [19]	178	IIIC–IV	3 or 4	PDS 47.6 NACT/IDS 67	PDS 41 NACT/IDS 43	PDS 15 NACT/IDS 14
TRUST [9]	688	IIIB–IVB	3	PDS 70 NACT/IDS 85	PDS 54.3 NACT/IDS 48.3	PDS 22.2 NACT/IDS 19.7

PDS, primary debulking surgery; NACT, neoadjuvant chemotherapy; IDS, interval debulking surgery; OS, overall survival; PFS, progression-free survival.

**Table 3:** Studies reporting no direct impact of the number of NACT cycles on oncologic outcomes.

Study (Author, Year)	Number of Patients	Cycles Compared	Outcomes (PFS/OS/Key Findings)
Stoeckle et al., 2011 [26]	137	≤4 vs. ≥5	No significant impact on outcomes
da Costa Miranda et al., 2014 [27]	82	6 cycles	Safe and effective; prolonged chemo did not worsen outcomes
Iwase et al., 2015 [28]	124	≤4 vs. ≥5	No significant impact on outcomes
Akladios et al., 2016 [29]	204	≤4 vs. ≥5	No significant impact on outcomes
Stewart et al., 2016 [30]	156	≤3 vs. ≥4	No significant impact on outcomes
Chung et al., 2017 [31]	197	≤3 vs. ≥4	No significant impact on outcomes
Phillips et al., 2018 [32]	398	≤4 vs. ≥5	No significant impact on outcomes if complete cytoreduction achieved after ≥5 cycles
Yoneoka Y et al., 2019 [33]	95	≤4 vs. ≥5	No significant impact on outcomes; selected patients may benefit from additional chemotherapy
Zorzato et al., 2020 [34]	108	≤4 vs. ≥5	No significant impact on outcomes
Yao et al., 2020 [35]	572	≤4 vs. ≥5	No significant impact on outcomes
Gupta et al., 2020 [36]	100	≤3 vs. ≥4	No significant impact on outcomes
Lecointre et al., 2020 [37]	200	≤4 vs. ≥5	No significant impact on outcomes; survival seems to be dependent on optimal resection and response to chemotherapy
Plett et al., 2020 [38]	308	≥5 cycles	Survival appears to be mainly determined by complete resection
Marchetti et al., 2021 [39]	315	≤4 vs. ≥5	No significant impact on outcomes
Nitecki et al., 2021 [40]	265	≤4 vs. ≥5	No significant impact on outcomes; residual disease was associated with worse survival outcomes
Bacry et al., 2022 [41]	140	3 vs. ≥4	No significant impact on outcomes; surgical morbidity is significantly reduced by increased cycles of chemotherapy
Betrian et al., 2022 [42]	365	≤4 vs. ≥5	No significant impact on outcomes
Perrone et al. 2023 [43]	286	≤4 vs. ≥5	No significant impact on outcomes, as long as complete surgical resection is achieved

OS, overall survival; PFS, progression-free survival; NACT, neoadjuvant chemotherapy.

**Table 4:** Studies reporting negative direct impact of the number of NACT cycles on oncologic outcomes.

Study (Author, Year)	Number of Patients	Cycles Compared	Outcomes (PFS/OS/Key Findings)
Bristol and Chi, 2006 [45]	835	Different studies, different NACT numbers (Meta-analysis)	Negative survival effect of increasing number of chemotherapy cycles
Colombo et al., 2014 [46]	147	≤4 vs. ≥5	Patients receiving complete surgery after more than 4 cycles had poor prognosis
Altman et al., 2017 [47]	403	≤3 vs. ≥4	>4 cycles of neoadjuvant chemotherapy were associated with a poorer overall survival
Bogani et al., 2017 [48]	193	≤3 vs. ≥4	Worse OS in patients receiving at least 4 cycles
Liu et al., 2020 [20]	199	≤4 vs. ≥5	Patients receiving ≥5 cycles may have worse prognosis, despite maximal cytoreduction
Minareci et al., 2022 [49]	221	≤3 vs. ≥4	Patients who received more than 3 cycles of NACT had poor OS; however, there was no statistical difference in terms of DFS
Thomas et al., 2022 [50]	2059	≤4 vs. ≥5	Patients treated by IDS after ≥5 cycles of NACT present worse survival outcomes
Gaba et al., 2025 [51]	2498	≤4 vs. ≥5	Women receiving >4 NACT had poorer OS, despite complete surgery

OS, overall survival; PFS, progression-free survival; NACT, neoadjuvant chemotherapy; DFS, disease-free survival; IDS, interval debulking surgery.

## Data Availability

No new data were created or analyzed in this study.
